# Effects of size and topology of DNA molecules on intracellular delivery with non-viral gene carriers

**DOI:** 10.1186/1472-6750-8-23

**Published:** 2008-02-29

**Authors:** Charlie Yu Ming Hsu, Hasan Uludağ

**Affiliations:** 1 Department of Biomedical Engineering, Faculty of Medicine and Dentistry, University of Alberta, Edmonton, Alberta, T6G 2G6, Canada; 2 Department of Chemical and Materials Engineering, Faculty of Engineering, University of Alberta, Edmonton, Alberta, T6G 2G6, Canada; 3 Faculty of Pharmacy & Pharmaceutical Sciences, University of Alberta, Edmonton, Alberta, T6G 2G6, Canada

## Abstract

**Background:**

Efforts to improve the efficiency of non-viral gene delivery require a better understanding of delivery kinetics of DNA molecules into clinically relevant cells. Towards this goal, three DNA molecules were employed to investigate the effects of DNA properties on cellular delivery: a circular plasmid DNA (c-DNA), a linearized plasmid DNA (l-DNA) formulated by single-site digestion of c-DNA, and smaller linear gene cassette generated by PCR (pcr-DNA). Four non-viral gene carriers were investigated for DNA delivery: polyethyleneimine (PEI), poly-*L*-Lysine (PLL), palmitic acid-grafted PLL (PLL-PA), and Lipofectamine-2000™. Particle formation, binding and dissociation characteristics, and DNA uptake by rat bone marrow stromal cells were investigated.

**Results:**

For individual carriers, there was no discernible difference in the morphology of particles formed as a result of carrier complexation with different DNA molecules. With PEI and PLL carriers, no difference was observed in the binding interaction, dissociation characteristics, and DNA uptake among the three DNA molecules. The presence of serum in cell culture media did not significantly affect the DNA delivery by the polymeric carriers, unlike other lipophilic carriers. Using PEI as the carrier, c-DNA was more effective for transgene expression as compared to its linear equivalent (l-DNA) by using the reporter gene for Enhanced Green Fluorescent Protein. pcr-DNA was the least effective despite being delivered into the cells to the same extent.

**Conclusion:**

We conclude that the nature of gene carriers was the primary determinant of cellular delivery of DNA molecules, and circular form of the DNA was more effectively processed for transgene expression.

## Background

Non-viral delivery systems are being pursued to facilitate therapeutic gene transfer in a clinical setting. Non-viral carriers, such as cationic lipids and polymers, typically interact with the anionic DNA via charged moieties, and condense the long, string-like DNA molecules into compact, nano-sized particles that are suitable for cellular uptake [1,2]. A range of cationic lipids and polymers has been engineered to accomplish this function effectively, and intelligent carriers are being continually designed with the purpose of controlling intracellular fate of DNA molecules. Most studies on non-viral gene transfer were conducted with circular plasmid DNA (c-DNA), which is known to be less susceptible to intracellular degradation. Linearized forms of c-DNA (l-DNA) with similar molecular weight, as well as shorter l-DNA that bears only the gene of interest and the promoter region have been used in some studies [3-8]. A c-DNA exhibits a higher intracellular diffusivity compared to its linearized forms [9], which facilitates its nuclear targeting for more effective expression. l-DNA is expected to be prone to nuclease attack intracellularly, but its stability could be increased by capping the 3' and 5' ends of the molecule with hairpin structures [8]. Smaller l-DNA molecules, on the other hand, displays better ability to traverse the nuclear membrane, potentially contributing to better translation of the transgene. Studies attempting to directly compare the efficiency of different DNA molecules yielded conflicting results. Whereas Cherng et al. observed better expression of *LacZ *gene when cells were transfected with c-DNA [9], Schakowski et al. observed a similar expression level (based on % transfected cells) for the two DNA topologies with both polymeric and lipid-based carriers [7]. A difference in the level of gene expression (based on IL-2 expression), however, was noted in the latter study, shorter l-DNA giving enhanced expression [7]. In direct nuclear injection studies, with no nuclear transport barriers to DNA molecules, l-DNA yielded more effective gene expression, suggesting an intrinsic superiority of l-DNA for transcription [6]. Shorter l-DNA molecules with intact promoter/gene regions were even more effective [6], possibly due to better assembly of transcriptional factors and/or reduced non-specific interactions of proteins with the DNA molecules. The nature of delivery vehicle appeared to influence the transfection efficiency, l-DNA being more sensitive to the choice of the carrier, unlike the c-DNA [8]. This was indicative of the differences in the transport efficiency among the carriers, but no studies directly compared the efficiency of cell delivery of different DNA molecules.

**Figure 1:**
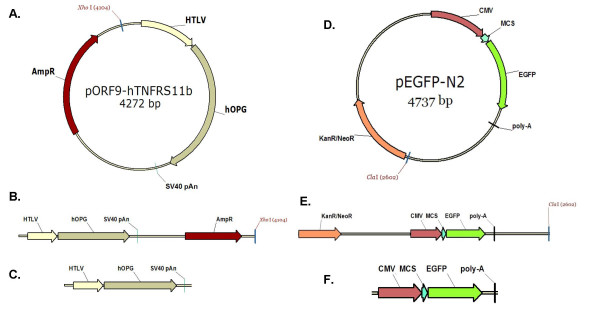
Structure of the DNA molecules used in this study. **A**. Structure of the parent c-DNA. **B**. l-DNA formed by restriction digest of c-DNA with the enzyme *Xho*I. The size of the linearized plasmid was 4272 bp, **C**. pcr-DNA (2209 bp) generated by site-specific primers that amplified the region of the c-DNA containing the HTLV promoter, the hOPG open reading frame, and the SV40 Poly-A site. **D**. Structure of the parent c-DNA used in transfection. **E**. l-DNA formed by digest of pEGFP-N2 with the enzyme *Cla*I, which cuts in the vector backbone. **F**. pcr-DNA (1713 bp) generated by primers that are complementary to the sequence upstream of the CMV promoter and downstream of the polyA site.

This study investigated the non-viral delivery of 3 types of DNA molecules (Figure 1), namely a c-DNA representing a circular plasmid, a linearized version of the c-DNA after restriction digest at a single site, and a shorter version of the l-DNA amplified by a PCR reaction (pcr-DNA). Four different non-viral carriers were utilized, including 2 cationic polymers commonly used for DNA delivery, poly-ethyleneimine (PEI) and poly-L-lysine (PLL), an in-house synthesized palmitic acid-grafted PLL (PLL-PA) [10], and the most-commonly used commercial lipid Lipofectamine-2000™. By using combinations of different DNA molecules and carriers, the influences of DNA molecular weights and topologies on the delivery to bone marrow stromal cells were investigated. These cells, unlike commonly used immortal cells, are an important cell phenotype for clinical protocols. Finally, transgene expression by the three DNA forms were investigated by using the reporter gene Enhanced Green Fluorescent Protein (EGFP). Our results indicated the delivery of all three types of DNA molecules was equally effective for each carrier, with significant carrier-to-carrier differences in delivery rates, but c-DNA were more effective in yielding reporter gene expression as compared to the linearized DNA molecules.

## Methods

### Materials

Branched 25-kDa PEI, PLL (25 kDa), Hank's Balanced Salt Solution (HBSS), trypsin/EDTA, and sodium salt of Ampicillin were obtained from SIGMA (St. Louis, MO). Lipofectamine2000™ was purchased from Invitrogen (Carlsbad, CA). Dulbecco's Modified Eagle Medium (DMEM; high glucose with L-glutamine), Penicillin (10,000 U/ml), and Streptomycin (10,000 μg/ml) were from GIBCO (Grand Island, NY). Dialysis tubing with a MW cut-off of 12-14 kDa was purchased from Spectrum Laboratories (Gardena, CA). Fetal Bovine Serum (FBS) was from Atlanta Biologicals (Lawrenceville, GA). A succinimide ester of Cy5.5 (Cy5.5-NHS) used for labeling DNA was purchased from Amersham (Baie d'Urfé, QC). PLL modified with palmitic acid was synthesized as previously described [10]. The plasmid gWiz-blank (5.1 kb), which lacks EGFP, was obtained from Aldevron (Fargo, ND).

### Preparation of Different DNA Molecules (Figure 1)

#### Circular Plasmid DNA (c-DNA)

The plasmid, pORF9-hTNFRS11b (4272 bp) encoding the human Osteoprotegerin gene, was purchased from Invivogen (San Diego, CA) as transformed *Escherichia coli *frozen culture. The plasmid, pEGFP-N2 (4737 bp), incorporating an enhanced green fluorescent protein was obtained from BD Biosciences. Cells were revitalized and grown in 12 L of Luria-Bertani broth containing either 100 μg/mL of Ampicillin (pORF9-hTNFRS11b) or 50 μg/ml of Kanamycin (pEGFP-N2) for 16 hours at 37°C with shaking at 125 rpm. Cells were harvested and plasmid was purified using QIAGEN Plasmid Giga Kit (Qiagen; Mississauga, ON) as described in the manufacturer's handbook. The purified plasmid was dissolved in ddH_2_O at a final concentration of ~1.8 mg/mL. Concentration and purity were determined by UV Spectroscopy using NanoDrop1000 instrumentation.

#### Linear DNA (l-DNA)

Purified c-DNA was linearized using the restriction enzyme, *Xho*I (New England Biolabs; Pickering, ON) for pORF9-hTNFRS11b or *Cla*I (Invitrogen; Burlington, ON) for pEGFP-N2, Restriction digestion were set up with 5 μg of DNA per 50 μL of reaction volume containing 3 units of enzyme and incubated at 37°C for 16 hours. The enzyme was then heat-inactivated by incubating the mixture at 65°C for 10 min. Digested DNA was purified using QIAEX II Gel Extraction Kit (Qiagen, Mississauga, ON). Purity of the isolated product was confirmed by spectrophotometry and gel electrophoresis was used to confirm the presence of a single band in the preparation.

#### Gene Cassette (pcr-DNA)

The primers, hOPG-F2 (5'-CGAAACAAAACAAACTAGCAAAAT-3') and hOPG-R2 (5'-CCTTTTGCTCACATGTTCTTAATTA-3') was used to synthesize gene cassette from the plasmid pORF9-hTNFRS11b; GFP-F1 (5'-TCCTGCGTTATCCCCTGATT-3') and GFP-R1 (5'-CGCTTACAATTTACGCCTTAAG-3') was used to synthesis gene cassette from the plasmid pEGFP-N2. All primers were customarily designed and synthesized by IDT Technology (Toronto, ON). Synthesis of gene cassette was carried out using PCR in a 50 μL reaction containing 0.6 μM of each of the primers, 0.2 mM dNTP, 1 unit of Platinum^® ^Taq DNA Polymerase High Fidelity (Invitrogen), 1× Hi Fi PCR buffer, 1.5 mM of MgSO_4_, and 20 ng of c-DNA as the template. Reaction parameters were as follows: 2 min at 94°C followed by 35 cycles of 30 s at 94°C, 30 s at 60°C, and 3 min at 68°C, then an addition 5 min incubation at 68°C. The PCR products so obtained were purified by QIAGEN PCR purification kit (Qiagen, Mississauga, ON). Purity of the isolated product was confirmed by spectrophotometry, and gel electrophoresis was used to confirm the presence of a single band in the preparation. The resulting PCR product was approximately 2209 bp (pORF9-hTNFRS11b) or 1684 bp (pEGFP-N2).

### Atomic Force Microscopy (AFM)

Prior to AFM measurements, carriers were added to the DNA solutions in 1:1 carrier-to-DNA weight ratios, and allowed to incubate for 20 min at room temperature. The solutions were diluted ×100 with ddH_2_O and 1.5 μL of the resulting solution was deposited onto freshly cleaved mica surfaces. Samples were allowed to dry in air for 20 min or until water had visibly evaporated from the surfaces. Experiments were performed with MFP-3D-BIO (Asylum Research; Santa Barbara, CA) operating in tapping mode (AC) using standard silicon cantilever (AC240TS) with ~2 N/m spring constant and 70 KHz working frequency. All images were recorded in air at room temperature inside an acoustic enclosure, and on a vibration isolation table at a scan speed of 1 Hz.

### Particle Size Measurement

The diameter of the particles formed by carrier/DNA complexes was measured by photon correlation spectroscopy (ZetaPALS, Brookhaven Instruments, Holtzville, NY). Four microgram of DNA was complexed with the appropriate volume of polymer solution at a carrier/DNA weight ratios of 5, 7.5 and 10 in a total volume of 50 μL diluted with 150 mM NaCl. The complexes were left to stand at room temperature for 30 minutes, then diluted to 2 mL with DMEM and incubated for another 30 minutes before analysis. The measurement time was set at 30 seconds intervals and each run consisted of 10 consecutive measurements (SD among the measurements was typically ~1%). Particle sizes were measured at a wavelength of 660 nm and calculated by using a medium viscosity of 1.140 cP [11] and a refractive index of 1.333 (at 25°C).

### Electrophoretic Gel Mobility Assay (EMSA)

Electrophoretic mobility of carrier/DNA solutions was performed by loading the complexes (see preparation condition below) into a 0.7-0.8% agarose gel containing 1 μg/mL of Ethidium Bromide in 1× Tris-Acetate/EDTA buffer. The agarose gel was run with 115 V of current for ~20 min and the DNA bands were visualized using the Alpha Imager (Alpha Innotech; San Leandro, CA). Mean fluorescent density (in arbitrary units) of each band was measured by the manufacturer-supplied software.

#### Analysis of Carrier-DNA Binding

200 ng of DNA was first suspended in 100 mM HEPES buffer (pH 5.2), carriers were then added to DNA solutions at DNA:carrier weight ratios of 2, 1, 0.5, 0.2, 0.1, and 0 in a final volume of 15 μL, and the solutions were allowed to incubate at room temperature for 30 min. The samples were then loaded into the gel as described above. The amount of free DNA in each lane was quantitated by densitometric analysis. The percentage of DNA bound was calculated from the fluorescent density (**F**) values as: % DNA bound = {**F**(DNA only) - **F**(specific DNA-polymer ratio)} ÷ {**F**(DNA only) - **F**(background)}•100%. %DNA bound vs. carrier:DNA ratios were plotted, and used to obtain a carrier/DNA-specific IC_50 _value, indicating the carrier:DNA ratio that gave binding to 50% of the DNA in the sample.

#### Analysis of Carrier-DNA Complex Dissociation

350 ng of DNA molecules in 150 mM NaCl were complexed with 3 μg of polymer in a final volume of 10 μL for 30 min at room temperature. Then, 10 μL of either basic medium (with 10% FBS) or 10 μL of DMEM was added to the samples, and incubated at room temperature for an additional 1.5 hours. Heparin sulfate were then added at a final concentration range of 0.02-32 μg/mL and the samples were incubated for 30 min. The samples were then loaded onto the gel and visualized as described above. The percentage of DNA dissociated was calculated from the fluorescent density (**F**) values as: %DNA released = {**F**(specific heparin concentration) - **F**(background)} ÷ {**F**(DNA only) - **F**(background)}•100%.

### Cell Culture and DNA Uptake

Rat bone marrow stromal cells (rBMSC) were isolated and cultured as described previously [12]. Briefly, cells were isolated from both femurs of 8-week old female Sprague-Dawley rats and pooled to obtain a single suspension. The bone marrow was flushed out with 15 mL of DMEM containing 10% FBS, 50 μg/mL ascorbic acid, 100 U/mL Penicillin and 100 μg/L of Streptomycin (referred to hereon as basic medium). Cells were centrifuged for 6 min at 600 rpm, suspended in fresh basic medium and seeded in a single 75 cm^2 ^flask (Sarstedt; Montreal, QC). After medium change on day 3, the cells were trypsinized on day 7 and expanded in 75 cm^2 ^flasks (1:4 dilution). The rBMSC passaged between 2-4 generations were used in this study, and were grown in multiwell plates for DNA uptake studies.

To investigate DNA uptake, 20 μg of DNA was labeled with Cy5.5-NHS as suggested by manufacturer's protocol. Labeling reaction was stopped by the addition 100 mM Tris-Cl to quench the pre-activated succinimide ester moiety. Labeled DNA was dialyzed against 10 mM Tris-Cl (pH 7.4) then again with ddH_2_O. The samples for uptake were prepared with 5 μL of the labeled DNA solution added to 2, 6 or 18 μg of the polymer (0.3, 1 and 3 μg for branched PEI) in a final volume of 40 μL. The samples were added directly to the rBMSC grown in 6-well plates with 2 mL of basic medium. Each polymer concentration was typically tested in duplicates. Cells are incubated for 24 hours at 37°C (5% CO_2_), after which they were washed with HBSS (x2), trypsinized for 5 min for detachment and suspended in HBSS with 4% formalin. Fluorescence was measured at λ_em _= 690 nm and λ_em _= 705 nm. The results were expressed as the percentage of cells exhibiting significant fluorescence over that of control samples (i.e. cells exposed to DNA with no polymer), which was set to ~1% uptake. In same cases, the uptake was expressed as the average fluorescence exhibited in cells (in arbitrary units).

### Transfection and Assessment of EGFP Expression

Two cell types were used for assessment of transgene expression, primary rBMSC and immortalized HEK 293T cells. BMSC were cultured and maintained as described in the DNA uptake studies. HEK 293T cells were seeded in 12-well tissue culture plates supplemented with DMEM media containing 10% FBS. When cells reached ~80% confluency, they were transfected with PEI/DNA complexes prepared at a polymer/DNA weight ratio of 2.5 (PEI:DNA concentration of 5:2 μg/mL in tissue culture medium). Complexes were incubated at room temperature for 25 minutes then added directly to cells covered in a basic medium. The cells were allowed to incubate at 37°C in the presence of complexes for 24 h followed by replacement of 1 mL of DMEM containing 10% FBS. Transfected 293 cells were either processed for flow cytometry following complex removal (24 hours), or incubated for another 72 hours before assessment of EGFP expression by flow cytometry. BMSC were processed 48 hours after complexes were removed. Flow cytometry was performed on a Cell Quanta SC with MPL Option (Beckman Coulter) where the EGFP fluorescence was detected in the FL1 channel. The instrument settings were calibrated for each run so as to obtain a background level of EGFP expression of ~1% for control cells. The latter included (i) cells treated with 2 μg/mL of c-DNA, l-DNA and pcr-DNA without any carriers, and (ii) cells treated with PEI/gWiz-blank plasmid that contained no functional genes (to ensure that internalized complexes do not provide a non-specific fluorescence in flow cytometry).

### Statistical Analysis

Where indicated, all results are summarized as mean ± standard deviation, and statistical variations (p < 0.05) between the group means were analyzed by the Student's *t*-test.

## Results

### Particle Formation

**Figure 2:**
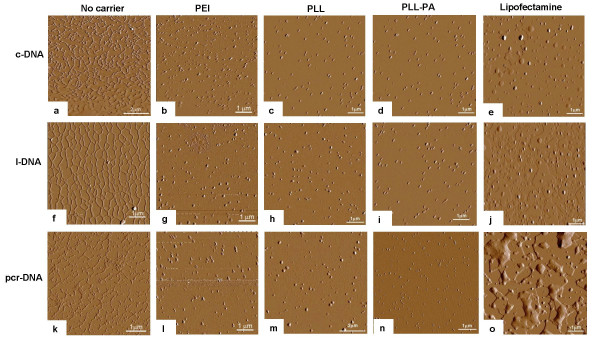
Tapping mode atomic force microscope amplitude topography of the three DNA molecules complexed with various gene carriers. All images were captured at a scan size of 6 × 6 μm, except where noted. c-DNA without carriers [8 × 8 μm] (**a**), with PEI (**b**), PLL (**c**), PLL-PA (**d**), and Lipofectamine-2000™ (**e**). l-DNA without carriers (**f**), with PEI (**g**), PLL (**h**), PLL-PA (**i**), and Lipofectamine2000™ (**j**). pcr-DNA without carriers [5 × 5 μm] (**k**), with PEI (**l**), PLL (**m**), PLL-PA (**n**), and Lipofectamine-2000™ (**o**).

The three DNA molecules were mixed with various gene carriers in solution to examine particle formation and morphology (Figure 2). When DNA molecules were examined under AFM in the absence of carriers, a network of interconnecting fibrous strands was observed, especially for c-DNA (Figure 2a) and l-DNA (Figure 2f), whereas pcr-DNA appeared as discontinuous strands (Figure 2k). When branched PEI was added to the DNA solutions (Figure 2b, 2g, and 2l), spherical particles were observed for all three types of DNA molecules with no evidence of string-like DNA molecules. The sizes of the particles ranged from ~40 nm to ~180 nm in diameter, and were independent of the type of DNA molecules used. Similar observation was noted for particles obtained with PLL (Figure 2c, 2h, and 2m) and PLL-PA (Figure 2d, 2i and 2n). The sizes of the particles formed by the PLL and PLL-PA were equivalent to those formed by the branched PEI. The cationic lipid, Lipofectamine-2000™, also bound and condensed c-DNA and l-DNA into spherical particles (Figure 2e and 2j). However, the sizes of some of the particles were significantly larger, with some particles being as large as ~450 nm. Some particles were observed when pcr-DNA were mixed with Lipofectamine-2000™ (Figure 2o), but isolated islands of presumably Lipofectamine-2000™ formed over the mica surface covering some of the DNA molecules. Although we followed the manufacturer's instructions for DNA condensation in the latter case, excess lipid was apparently present in these solutions. At the same time, some string-like structures, reminiscent of naked pcr-DNA, were also seen in these samples, suggesting incomplete condensation of pcr-DNA molecules with the Lipofectamine-2000™. Taken together, all of the gene carriers examined was able to condense the c-DNA, l-DNA and pcr-DNA molecules into spherical particles, except the Lipofectamine-2000™/pcr-DNA combination, which gave incomplete condensation of the DNA molecules.

The particle sizes were additionally analyzed by dynamic light scattering (Table 1). Unlike AFM, this method characterizes the size of the particles in solution and it can be considered more representative of the state of the particles in contact with the cells. Three different carrier:DNA ratios were used to investigate its effect on particle sizes. Whereas some combinations (e.g., PLL-PA and PEI combined with c-DNA) gave the expected decrease in size with increasing carrier:DNA ratio, other combinations (e.g., ones involving l-DNA and Lipofectamine-2000™) did not give a predictable pattern. Particles from Lipofectamine-2000™ were generally larger for each DNA molecule, and, in some cases, no stable measurements could be taken either due to visible precipitation of the complex solution or formation of large particle aggregates. This was consistent with the AFM observations on Lipofectamine-2000™ complexed DNA molecules. With PLL-PA, l-DNA gave relatively larger particles (~350 nm) as compared to c-DNA and pcr-DNA (100-160 nm). This was the case with PEI as well, except PEI complexes appeared to be significantly larger than the PLL-PA complexed particles for each type of DNA molecule.

**Table 1:** Mean particle sizes (nm) obtained for the DNA molecules at three (5.0, 7.5 and 10.0) carrier:DNA mass ratios. The values shown are usually derived form the average of 2 measurements, and SD between the measurements was <5% (not shown). NS: No stable measurements were taken for these samples. The zeta potential of the particles were >30 mV in all cases (not shown).

	**Circular DNA**			**Linear DNA**			**PCR DNA**		
	
	**5.0**	**7.5**	**10.0**	**5.0**	**7.5**	**10.0**	**5.0**	**7.5**	**10.0**
**PEI**	337	188	159	735	710	772	518	414	273
**PLL-PA**	169	124	106	354	388	338	104	99	103
**Lipo**	758	964	1002	NS	1406	421	NS	NS	NS

### DNA Binding

**Figure 3:**
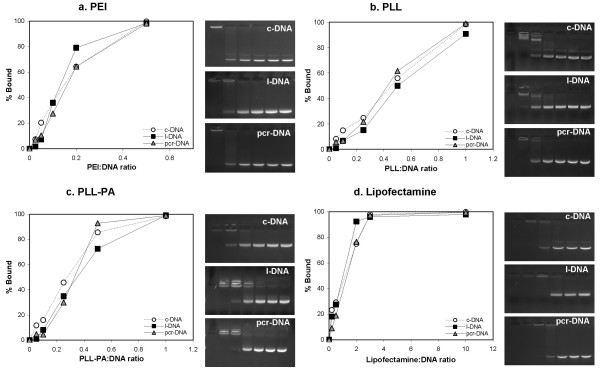
Analysis of DNA binding to the gene carriers PEI **(a)**, PLL **(b)**, PLL-PA **(c)**, and Lipofectamine-2000™ **(d)**. Densitometric analyses of the binding results in the form of percent DNA bound were summarized as a function of increasing carrier:DNA weight ratio. The corresponding gel pictures of DNA-carrier complex were also shown.

Figure 3 shows the results of the densitometric analysis from the EMSA assay intended for semi-quantitative analysis of DNA-carrier interactions. For each carriers examined, there was no significant difference in the binding interaction among the DNA molecules; i.e., the curve indicating percent bound vs. carrier:DNA ratio overlapped for all three DNA molecules. However, the carrier:DNA ratios necessary for complete (100%) binding and for IC_50 _were different among the carriers. Complete binding were achieved at ~0.5, ~1.0, ~1.0, and ~3.0 carrier:DNA ratios for branched PEI, PLL, PLL-PA, and Lipofectamine-2000™, respectively (Figure 3a, 3b, 3c, and 3d). The IC_50 _values were ~0.15, ~0.45, ~0.3, and ~1.2 for branched PEI, PLL, PLL-PA, and Lipofectamine-2000™, respectively.

### Complex Dissociation

**Figure 4:**
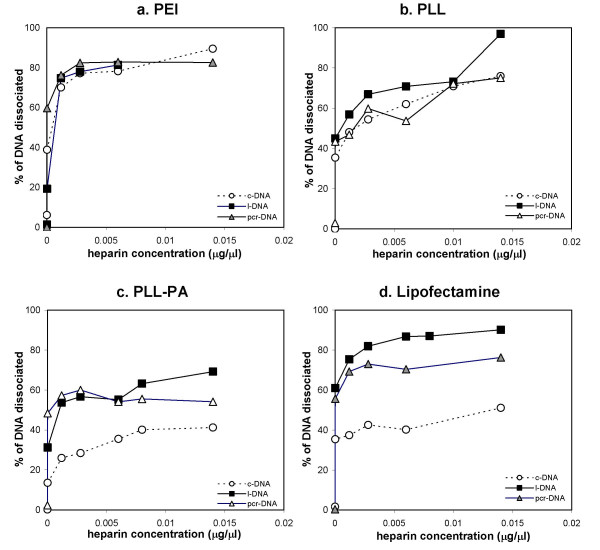
Dissociation kinetics of DNA-carrier complexes using heparin sulfate as the competitive polyanion. Results are displayed as a function of the concentration of heparin added and the percent of DNA released. Shown here are dissociation of the complexes with PEI **(a)**, PLL **(b)**, PLL-PA **(c)**, and Lipofectamine-2000™ **(d)**.

The dissociation of DNA/carrier complexes was investigated by incubating heparin sulfate with the complexes. The dissociation was conducted in complete culture medium to mimic the conditions while the complexes are incubated with the cells. Heparin was able to dissociate the complexes for all carriers (Figure 4), based on the liberation of free DNA molecules upon incubation with the highest heparin concentration. With branched PEI, the dissociation of the complexes as a function of heparin concentration was similar for all three DNA molecules (Figure 4a), where >80% dissociation of the complexes was observed at the highest heparin concentrations. This was the case for PLL as well (Figure 4b), except the extent of dissociation was generally less than 80%. With PLL-PA and Lipofectamine-2000™, c-DNA gave a relatively lower heparin-induced dissociation as compared to l-DNA and pcr-DNA (Figure 4c and 4d, respectively) and, in both cases, it was not possible to release more than 50% of the c-DNA.

### DNA Delivery to rBMSC

**Figure 5:**
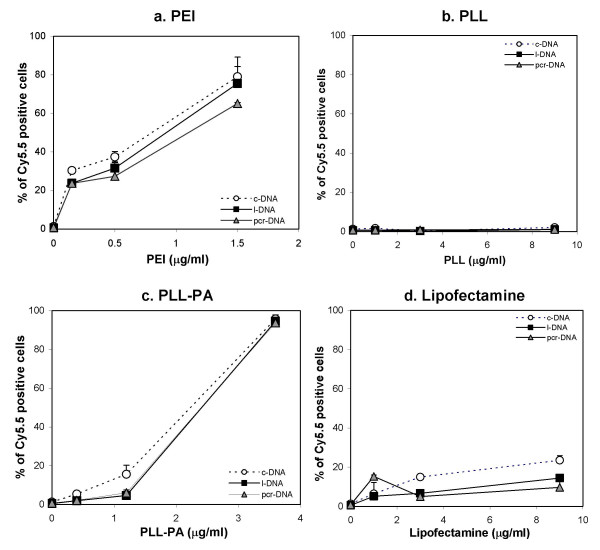
Delivery of c-DNA, l-DNA and pcr-DNA into rBMSC using PEI **(a)**, PLL **(b)**, PLL-PA **(c)**, and Lipofectamine-2000™ **(d) **at various concentrations of carriers. The cells were grown in 6-well plates for 24 hours, and incubated with carrier/DNA complexes for an additional 24 hours. Results from the flow cytometry were expressed as percentages of cells positive for Cy5.5-fluorescence, where the cells incubated with DNA alone (i.e., without any carrier) was calibrated at ~1%.

The intracellular DNA delivery was investigated by using DNA molecules fluorescently labeled with Cy5.5 fluorophore. Figure 5 shows the results of Cy5.5-labeled DNA uptake by BMSC grown in basic medium, which were carried out with a fixed concentration of DNA and varying concentrations of the carriers. In all cases, the cellular delivery increased with increasing carrier concentration in the formulations. The percentages of Cy5.5-positive cells were generally highest when branched PEI and PLL-PA were used as the carriers. Branched PEI was able to deliver all three types of DNA molecules up to ~80% of the cells at a lower polymer concentration as compared to the PLL-PA (~1.5 μg/mL vs. ~3.0 μg/mL; Figure 5a and 5c, respectively). PLL did not give any elevated level of DNA uptake in comparison to cells exposed to their respective DNA controls (Figure 5b). Lipofectamine-2000™ had a maximum of ~20% of Cy5.5-positive cells at the highest concentration tested (9 μg/ml; Figure 5d). For each carrier, there were no apparent differences in the delivery efficiency among the three DNA molecules.

### Effect of Serum on DNA Delivery

**Figure 6:**
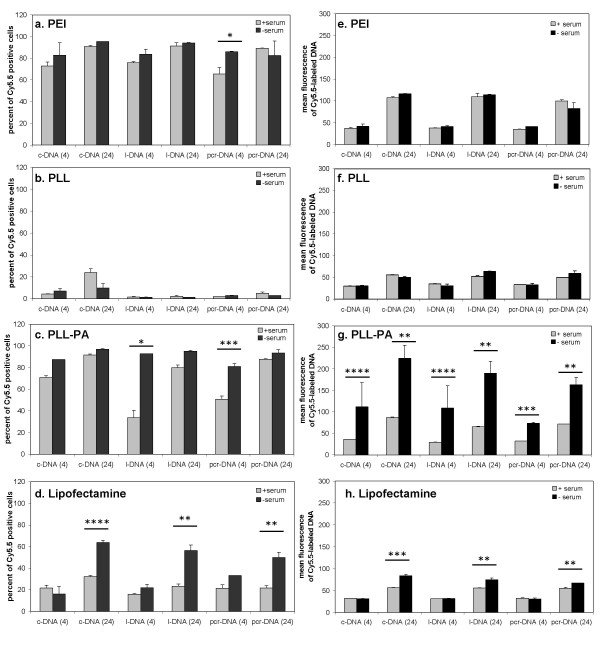
DNA delivery by PEI (a and e), PLL (b and f), PLL-PA (c and g), and Lipofectamine-2000™ (d and h) in the presence and absence of serum. The cells were grown in 24-well plates for 24 hours, and incubated with carrier/DNA complexes for an additional 4 and 24 hours. Results from the flow cytometry were expressed either as percentages of cells positive for Cy5.5-fluorescence (a, b, c and d) or mean fluorescence for Cy5.5-positive cells (e, f, g and h). Statistical differences between the medium with and without serum are indicated above: *p < 0.05, **<0.025, ***p < 0.01 and ****p < 0.001.

Several studies have shown serum in culture medium to influence gene delivery by non-viral gene carriers [13,14]. To investigate whether serum had an effect on delivery of different DNA molecules, complexes were incubated with the cells for 4 and 24 hours in the presence (10% serum) and absence of serum. Branched PEI and PLL-PA again gave the highest extend of DNA delivery (Figure 6a and 6c, **respectively**), with PLL giving the lowest delivery of DNA to the cells (Figure 6b), similar to the results shown in Figure 5. With branched PEI (Figure 6a), the extent of DNA delivery among the three DNA molecules was similar (70-90%). The presence of serum did not have a major effect on DNA delivery, except at the early time point (4 hour) for pcr-DNA, where the uptake appeared to increase by ~31% in the absence of serum. With PLL-PA as the carrier (Figure 6c), the extent of DNA delivery reached saturation (~100% of rBMSC) for the three types of DNA molecules in the absence of serum. However, l-DNA and pcr-DNA gave lower delivery at earlier time point (4 hour) in the presence of serum (~64% and ~37% reduction, respectively). With Lipofectamine-2000™ as the carrier (Figure 6d), the extent of delivery was low in the presence of serum (~20%) for all DNA molecules. In the absence of serum, DNA uptake after 24 hours was ~2-fold higher for all three DNA molecules.

The average fluorescence of Cy5.5-positive cells after incubation with complexes is also shown in Figure 6. These fluorescent values were used as a quantitative measure of the amount of DNA internalized by the rBMSC. Consistent with the percent uptake results, the highest DNA delivery was obtained with the branched PEI and PLL-PA as the carriers. With branched PEI, there was a ~2-fold increase (p < 0.01) in DNA delivery from 4 to 24 hour time point (Figure 6a). Neither serum nor the type of DNA molecules had an effect on the amount of Cy5.5-labeled DNA being internalized by the cells. When PLL was used as the carrier, the amount of internalized Cy5.5-labeled DNA was low; there was no effect of the serum or the type of DNA molecules on the DNA delivery (Figure 6b). With PLL-PA as the carrier, ~2-fold increase in the DNA uptake was again observed upon incubation of the complexes with the cells from 4 to 24 hours (Figure 6c). In this case, approximately 2.3-3.7 fold increase in DNA delivery was observed in the absence of serum with all three types of DNA molecules. With Lipofectamine-2000™, an increase in DNA delivery was also observed under longer incubation time (4 vs. 24 hours, Figure 6d). Eliminating serum gave a significant increase in DNA delivery for all three types of DNA molecules at the 24-hour time point.

### Transgene Expression

**Figure 7:**
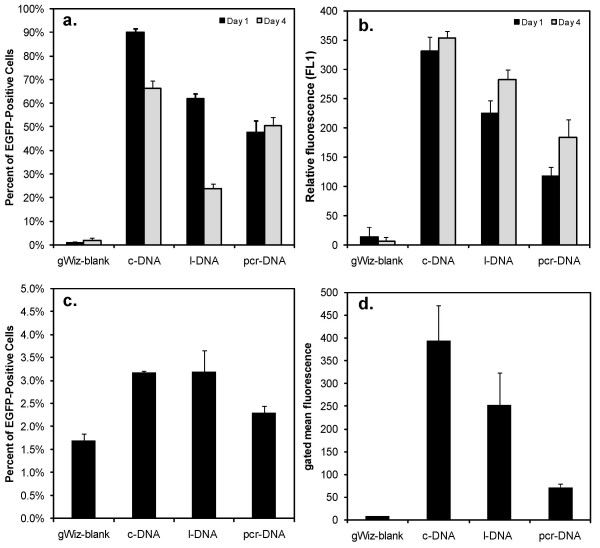
Transfection efficiency of c-DNA, l-DNA, and pcr-DNA using PEI as the gene carrier in HEK 293T Cells (**A **and **B**) and rBMSC (**C **and **D**). Cells were grown in 12-well plates and incubated with the complexes for 24 hours. EGFP expression was assessed on Day 1 and 4 for 293 cells, and on Day 3 for rBMSC. Results from the flow cytometry were expressed as either (i) percentage of EGFP-positive cells, when control cells were assigned to ~1% EGFP-positive cells (**A **and **C**), or (ii) the relative fluorescence values for EGFP-positive cells (**B **and **D**). The control cells in both cases were the cells exposed to PEI/gWiz-blank complexes without the EGFP gene. In the case of 293 cells, the relative fluorescence for the EGFP-positive cells was normalized with the fluorescence values for control cells to account for the day-to-day differences. For rBMSC, the mean fluorescence values were shown without normalization.

Assessment of gene expression was carried out by using the reporter gene EGFP, rather than the OPG plasmid previously used, since quantitating the extent of gene expression could be readily assessed by flow cytometry. Preliminary studies using pEGFP-N2 with PLL, PLL-PA, Lipofectamine-2000™ and PEI indicated the PEI to be the most effective carrier in our set up (data not shown). Therefore, subsequent expression studies were performed by using PEI as the sole carrier and the three types of DNA molecules to specifically focus on the relative effectiveness of each DNA molecule for the expression of reporter gene (rather than focusing on the effectiveness of each carrier). To determine if the enzymatic manipulations used to generate the l-DNA and pcr-DNA yielded functional gene constructs, transfections were initially carried out in HEK 293 T cells since they are considered to be a readily-transfectable cell phenotype. The results obtained from these cells are summarized in Figure 7. The c-DNA was the most effective form of DNA based of percentage of EGFP-positive cells (Figure 7a), giving ~90% cells with EGFP expression on Day 1. Transfection efficiency using l-DNA and pcr-DNA were significantly lower: ~61% and ~48% EGFP-positive cells, respectively. On Day 4, the percentage of EGFP-positive cells was reduced for c-DNA and l-DNA (but not pcr-DNA) transfected cells as compared to Day 1. The relative mean fluorescence values of the EGFP-positive cells (Figure 7b) were used as a measure of extent of EGFP expression; the c-DNA transfected cells displayed ~3.5 and ~2.5 fold increase in mean fluorescence over l-DNA and pcr-DNA transfected cells, respectively (Day 4). Non-treated cells, as well as the cells exposed to the three forms of DNA alone (i.e., without any carrier) gave results similar to the cells treated with PEI/gWiz complexes (not shown).

Using complexes similar to the ones used on 293 cells, transfection efficiency of rBMSC was found to be much lower; control cells exposed to PEI/gWiz complexes without an EGFP gene gave ~1.5% EGFP-positive cells as a background (Figure 7c), which was similar to values obtained with non-treated cells, and cells exposed to the three DNA molecules without any carrier (~1%; not shown). rBMSC transfected with complexes containing a functional EGFP gene afforded only 2-3% EGFP-positive cells (Figure 7c). There was no significant difference in the percent of GFP-positive cells between c-DNA and l-DNA; however, both forms of DNA were significantly more effective than pcr-DNA (p < 0.05). Among the EGFP-positive cells, the mean fluorescence values for all three DNA molecules were significantly greater than the control cells (Figure 7d). The cell treated with c-DNA had a greater mean fluorescence than the cells treated with l-DNA (p < 0.05), and both c-DNA and l-DNA gave a significantly (p < 0.05) greater mean fluorescence values than the pcr-DNA.

## Discussion

Non-viral gene delivery systems employ cationic polymers or lipids as vectors for delivery of therapeutic genes into pertinent cells. Previous studies have examined the effects of DNA molecular weight and topology on transfection efficiency [6,8] but failed to study the intracellular delivery of different DNA molecules *per se*. We employed three types of cationic polymers and one cationic lipid to determine if common non-viral gene carriers would have the same delivery efficacy using different physical forms of DNA. To compare the effects of physical properties alone on cellular uptake, all DNA molecules were manipulated from a single source of circular plasmid DNA extracted from bacteria. l-DNA and pcr-DNA were prepared by restriction digest and PCR, respectively, using c-DNA as the source or the template. The procedures were optimized to obtain homogenous products without gel electrophoresis. It is known that EtBr/UV light combination damages DNA by inducing strand breakage and oxidation [15,16]. To avoid this potential artifact, all DNA preparations have been performed in the absence of EtBr.

The ability of gene carriers to bind DNA and condense the molecule into particles is assessed qualitatively by AFM and quantitatively by photon correlation spectroscopy. AFM assessment indicated that the cationic polymers were able to condense all three types of DNA molecules into spherical particles with no discernible morphological differences. A variation in particle sizes was evident for individual carriers in AFM measurements, but size ranges appeared to overlap between the types of DNA molecules. Photon correlation spectroscopy indicated the PEI complexed DNA to be larger than the PLL-PA particles. The smaller size of the particles obtained from AFM is not surprising, considering that they were obtained under 'dry' condition which is likely to represent a collapsed state of the particles. The type of DNA molecule was a determining factor in the sizes of particles formed with the polymeric carriers (Table 1). Lipofectamine-2000™ also condensed DNA into spherical particles for both c-DNA and l-DNA, and the particles were similar in size to those observed elsewhere [17]. However, full condensation did not occur for the pcr-DNA at the weight ratios used in this experimental set-up. This was also the case for these particles from photon correlation spectroscopy measurements. Based on semi-quantitative EMSA analysis [18], no significant differences in the binding interaction among the three types of DNA molecules was evident for each carrier. Branched PEI was able to completely condense DNA at lower polymer-to-DNA weight ratio compared to other carriers. Both PLL and PLL-PA were able to completely condense DNA at the same ratio, however, the modification of PLL with palmitic acid led to an increase in binding affinity, contrary to the observation reported earlier [10]. The combination of AFM and EMSA results pointed to no apparent differences between the DNA molecules and their interaction with the carriers. It was interesting to note that EMSA results (indicating 100% binding at carrier:DNA ratios of ≤1), were mostly consistent with the AFM results, which indicated no free DNA at the carrier:DNA ratio of 1. The only exception was the results from Lipofectamine-2000™, where complete binding was seen at the carrier:DNA ratios of ~3, whereas AFM indicated no free DNA (for c-DNA and l-DNA) at the ratio of 1. It was possible that Lipofectamine-2000™ complexation led to more fragile particles that did not withstand the electrophoretic forces under EMSA assay conditions.

Dissociation of DNA from gene carriers is essential for efficient gene expression because access by the transcription machinery requires free and intact DNA [19,20]. The dissociation characteristics of the complexes were studied using heparin sulfate as a competitive polyanion to disrupt the interaction between the carriers and DNA molecules. Heparin sulfate is an anionic polysaccharide known to be a major component of extracellular matrix, and have been shown to inhibit DNA delivery [21-23]. It was used here to mimic the conditions that particles encounter under cell culture conditions. For DNA complexes formed with PEI and PLL, there was no significant difference in the dissociation profile among the three types of DNA molecules. With both vectors, however, DNA could not be fully dissociated from the complex. This may be due to a rate-dependent reorganization process that takes place after the initial complexes were formed, leading to a 'mature' complex with stronger electrostatic interaction that cannot be easily disrupted [19]. Complexes of PLL-PA and Lipofectamine-2000™ showed a different dissociation profile among the three types of DNA, where linear forms of DNA molecules were dissociated to a greater extent than the c-DNA. This suggested that a similar maturation process exist for the lipoplexes [24], and the extent of maturation varied among the different topologies of DNA. Grafting of the fatty acid palmitic acid on PLL renders a partial lipophilic characteristic to the cationic polymer, and is likely to account for the similarity in the dissociation profile to that of Lipofectamine-2000™, that is, with c-DNA being the least dissociated. Overall, our results suggested a similar efficiency in the unpacking of vector (dissociation) among the three DNA molecules for the polymeric carriers, but not for the lipid-based carriers whose dissociation was stronger with l-DNA molecules. Since expression of the transgene (OPG) was not explored in this study, it remains to be determined whether such a difference in dissociation would ultimately lead to differences in expression efficiency for different DNA isoforms.

With respect to DNA delivery to the cells, we observed no major difference in the level of DNA uptake among the three physical forms of DNA molecules. This was the case despite the fact that average particle sizes were found to depend on the nature of the DNA molecule. It is possible that significant variations in particle sizes in each complex preparation might have masked such a possible effect. Different routes of entry (i.e., clathrin- vs. caveolae-mediated) could also have been utilized by different complexes [25], but our studies were not designed to probe these differences. It must be pointed out that serum could not be used during photon correlation spectroscopy due to unstable readings obtained. Any changes in particle sizes in the presence of serum [26,27] might complicate our understanding of size effects on the DNA delivery. However, there was a difference in the delivery efficiency of DNA among the carriers. PEI was able to deliver DNA to cells at much lower concentrations and was the most effective carrier of the four examined. PLL was the least effective carrier for DNA uptake and its performance was comparable to when no carrier was used. In previous studies, PLL was shown to be readily taken up by the cells [28] but it appeared that its ability to carry a DNA cargo was relatively low. This is in contrast to the results obtained in other studies when fluorescent microscopy was used to detect DNA uptake [28]. The low level of uptake observed in this study may be attributed to the low sensitivity of the flowcytometer or a high cut-off level set up with the control samples (i.e. no carrier). There was no apparent correlation between particle formation, polymer-DNA interaction, complex dissociation and the DNA uptake. For example, PLL, despite showing typical DNA binding and dissociation activity, did not enable an equivalent DNA level of delivery into cells when compared to other carriers. This conclusion was valid for all three DNA molecules used in this study.

The presence of serum during cellular internalization of complexes did not influence the uptake of complexes of pure cationic carriers PEI and PLL, but did influence delivery by the lipophilic Lipofectamine-2000™ and PLL-PA. This may be due to serum inhibition of the complex maturation process, resulting in reduced uptake [24]. The fact that 24-hour uptake was still influenced by the presence of serum suggested the maturation process to last throughout this time period. Serum has previously been described to inhibit lipofection [29] as well as DNA uptake of lipoplexes [30,31]. For PLL-PA, only the linear forms of the DNA were affected by the serum. Given its partial lipophilic characteristic, the difference in the level of uptake may be directly related to the rate of complex maturation mentioned earlier. The observation that l-DNA is affected to a greater extent than pcr-DNA may be attributed to size differences between the molecules. Larger l-DNA was expected to take longer to reorganize itself into the matured complex; this may cause it to become more susceptible to binding by serum proteins than smaller l-DNA. All polymers resulted in an increase in absolute amount of DNA delivery from 4 to 24 hours, suggesting that the uptake pathway is a rate-limiting process for these carriers. Furthermore, PLL-PA showed a significant increase in absolute amount of DNA delivery in the absence of serum, where a similar increase was absent in Lipofectamine-2000™. Serum thereby affected PLL-PA differently than the Lipofectamine-2000™. The difference in the degree of sensitivity to serum can be accounted for by the nature of the lipid groups (monovalent vs. multivalent) [24], in addition to the polymeric nature of PLL-PA, which contributed to the continued uptake of complexes in the absence of serum.

The transfection results from both 293 cells and rBMSC showed that the supercoiled circular plasmid DNA was more effective topology for transfection than its linearized equivalent of the same molecular weight. This observation was consistent with previous reports [5,32]. Remaut et al. [5] attributed better translocation of c-DNA to perinuclear regions for better transgene expression by the c-DNA. We have further investigated the effectiveness of a gene cassette, which is approximately half the size of the linearized plasmid DNA; pcr-DNA was even less effective than l-DNA, even though the number of gene-encoding templates was expected to be twice that of l-DNA or c-DNA (per unit mass basis exposed to the cells). The carrier-DNA interactions (either binding or dissociation) were not expected to be different between the l-DNA and pcr-DNA at a given carrier:DNA ratio, as well as the uptake into the cells (data from Figures 3, 4 and 5). Thus, the difference in transfection efficiency between l-DNA and pcr-DNA cannot be explained from these considerations. The only difference between these two DNA molecules was in the hydrodynamic diameter of the complexes, where pcr-DNA gave smaller particles compared to l-DNA, but our data on this issue is too limited to probe a correlation between transfection and complex size. The difference observed in transfection could be attributed to intracellular response to the DNA itself. Several factors might come into play, including; (i) different rates of intracellular degradation of the DNAs (the smaller pcr-DNA could be more sensitive to degradation due to lower content of ectopic sites), and (ii) ease of accessibility of the template to transcription factors (the promoter of the smaller pcr-DNA might be more effectively blocked with the carrier as compared to the longer l-DNA). It is also possible that different types of DNA molecules would elicit different metabolic responses. For example, double-stranded l-DNA and pcr-DNA have exposed 5'-phosphate groups on their ends, which resemble damaged DNA. When introduced into cells, these DNA molecules might cause an inhibition of overall gene expression as a protection mechanism against aberrant expression, effectively reducing transgene efficiency. Still other factors may come into play, but this study was not intended to explore the intracellular fate and consequences of exogenous DNA delivery. Further studies on these issues will be conducted in the future to explain the difference in transfection efficiency among different DNA molecules.

## Conclusion

In conclusion, this study aimed at evaluating the delivery of different sizes and topologies of DNA by non-viral carriers. We have shown that there were no discernible differences in either the *in vitro *binding interaction to the gene carriers or the efficiency of uptake between either the circular and linear forms or between large and small DNA molecules. There was some effect on uptake and complex interaction in the presence of serum, but this difference was mostly influenced by the nature of the carrier itself, rather than the inherent properties of the DNA molecules. With the chosen PEI carrier, c-DNA was found to be more effective than the linearized DNA molecules for transgene expression. Better delivery of c-DNA into the cells was not the reason for its better performance, since the chosen carrier was able to efficiently deliver all DNA molecules into the cells regardless of their sizes and topologies. Intracellular processing of the DNA molecules was more likely to be the underlying reason for better efficiency of c-DNA.

## Authors' contributions

CYMH carried out all the experiments as outlined in the methods. HU was involved in the overall study design and in proposing appropriate directions during the daily execution of these studies. Both authors were involved in the drafting and revision of the final manuscript.
